# Fluorescence2D: Software for Accelerated Acquisition and Analysis of Two-Dimensional Fluorescence Spectra

**DOI:** 10.1371/journal.pone.0101227

**Published:** 2014-07-01

**Authors:** Evgenii L. Kovrigin

**Affiliations:** Chemistry Department, Marquette University, Milwaukee, Wisconsin, United States of America; National Research Council of Italy, Italy

## Abstract

The *Fluorescence2D* is free software that allows analysis of two-dimensional fluorescence spectra obtained using the accelerated “triangular” acquisition schemes. The software is a combination of Python and MATLAB-based programs that perform conversion of the triangular data, display of the two-dimensional spectra, extraction of 1D slices at different wavelengths, and output in various graphic formats.

## Introduction

Measurements of fluorescence are widely applied in biochemical sciences and biotechnology [1]. Analysis of biochemical samples often requires observation of multiple fluorophores [2], [3]. To enable analysis of complex fluorophore mixtures and reactions, the two-dimensional fluorescence measurements have been described [4], [5] where the excitation wavelength is systematically scanned while the corresponding emission spectra are collected. The result is a two-dimensional correlation map enabling identification of multiple fluorophores in the mixture, their interactions, and the time-dependent evolution. Practical usefulness of the two-dimensional spectra was exemplified by investigations of nucleotide exchange in Ras GTPase [6], analysis of structural details of DNA and RNA constructs [7] as well as real-time process monitoring [8]–[10].

**Figure 1:**
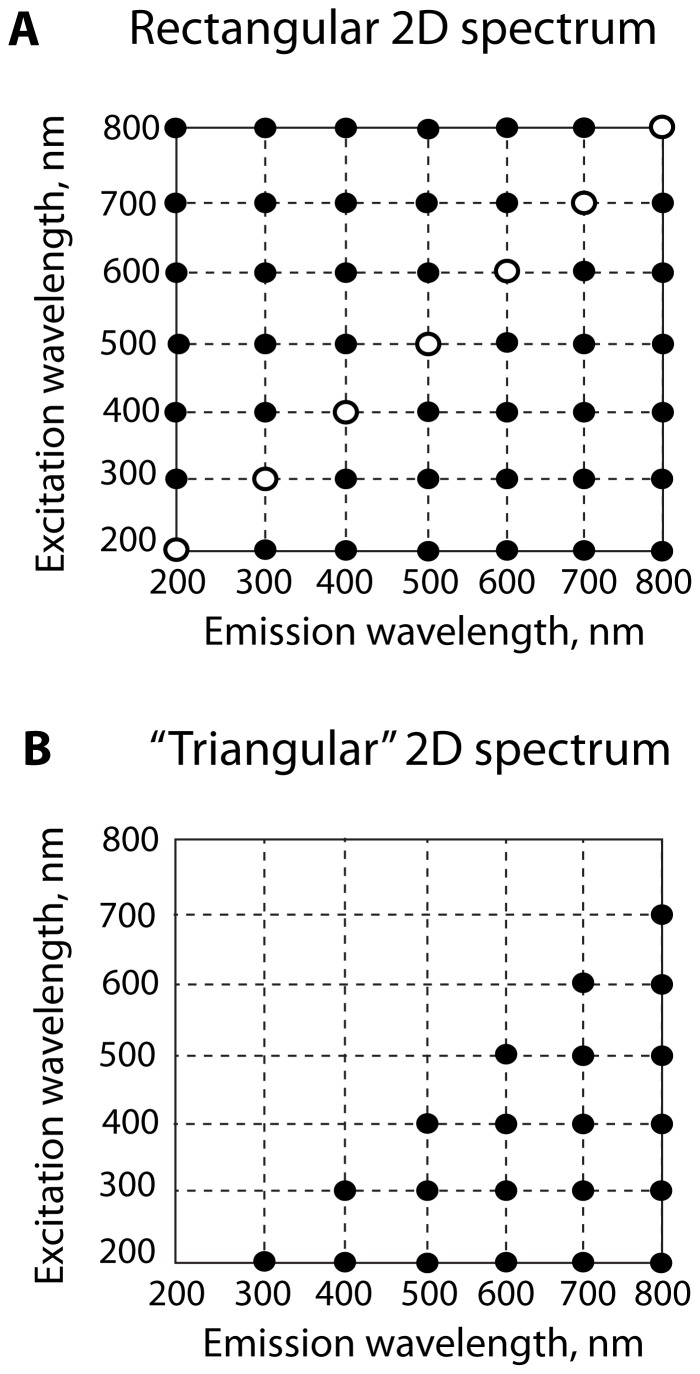
Rectangular and triangular two-dimensional spectra. (**A**) Example of a conventional array of excitation/emission wavelengths used for acquisition of two-dimensional fluorescence spectra. The diagonal (λ_em_ = λ_ex_) is shown with the open symbols. The conventional 1D emission spectrum is a horizontal set of points for each excitation wavelength to the right of the open symbol. Correspondingly, the 1D excitation spectrum is a vertical set of points below the open symbol. (**B**) The triangular array of excitation/emission wavelengths where the emission ranges always begin at a greater wavelength than the excitation wavelength in each row. The specific 100 nm increment in this figure is chosen solely for clarity of presentation.

**Figure 2:**
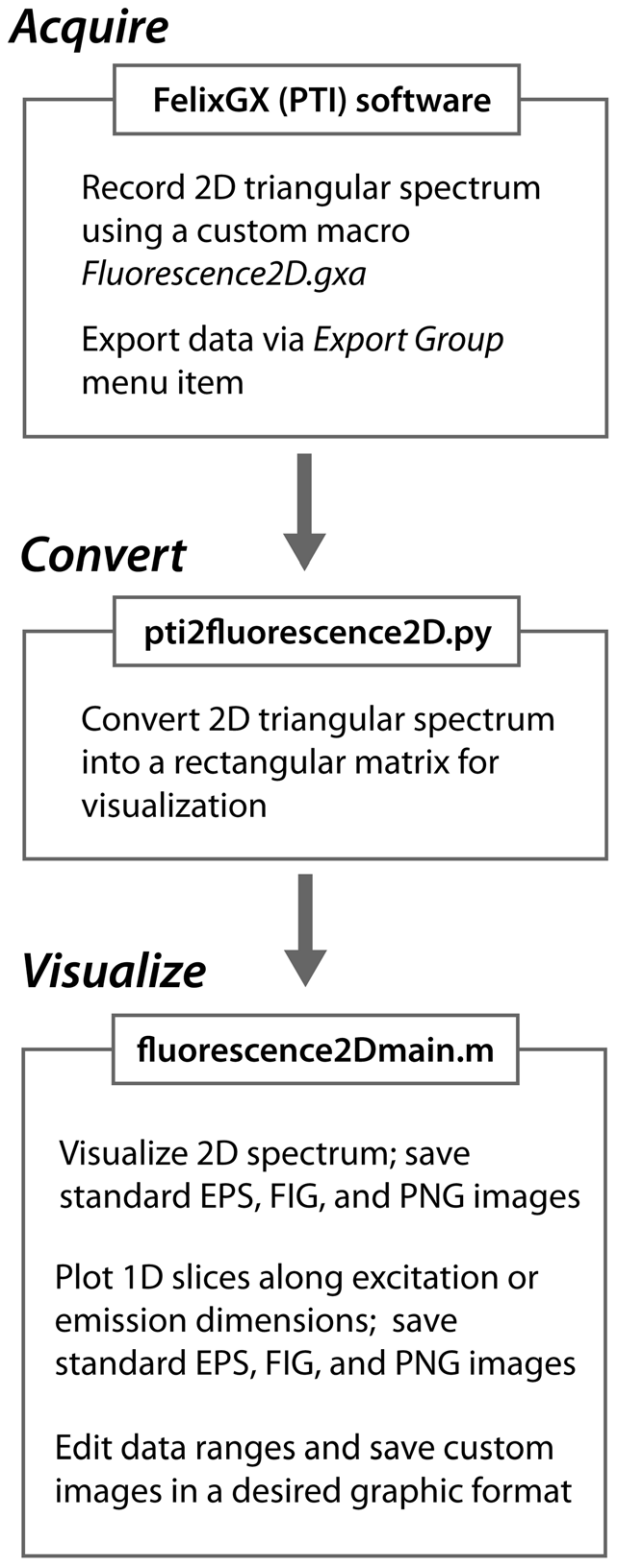
A typical workflow of the two-dimensional acquisition and data analysis.

A significant problem hindering applications of the two-dimensional acquisition is that the existing commercial software packages require the 2D data acquired as a *rectangular* array where the full emission spectral range is acquired for each value of the excitation wavelength (Figure 1.A). As a result, acquisition of such “rectangular” spectra wastes the instrument time because the emission spectra at the wavelengths *shorter* than the current excitation wavelength are empty at typical (relatively low) intensities of the commercial Xenon light sources. It is more practical to record the emission range only for the *longer* wavelengths than the current excitation wavelength that will result in “triangular” datasets (Figure 1.B). This mode of operation results in up to two-fold time saving allowing faster acquisition of the 2D spectra. To enable display and analysis of triangular spectral matrices, which cannot be visualized using commercial software, I developed the *Fluorescence2D* software package implemented in Python and MATLAB (Mathworks). *Fluorescence2D* is freely available for download from http://lineshapekin.net/#Fluorescence2D. The present version of *Fluorescence2D* is designed to work with the data obtained on PTI fluorometers (Photon Technology International, Canada); compatibility with other data formats will be added in the future. A typical workflow for acquisition and analysis of the two-dimensional fluorescence data is shown in the Figure 2.

## Algorithms and Implementation

### Acquisition of triangular 2D data

The key timesaving trick in acquisition of 2D fluorescence datasets is to reduce the emission wavelength range at every step when the excitation wavelength is incremented (Figure 1.B), which results in a triangular matrix, for example:

Row 1: excitation at 200 nm; scan emission range 210–800 nm with 10 nm step;

Row 2: excitation at 210 nm; scan emission range 220–800 nm with 10 nm step;

Row N: excitation at 790 nm; emission point at 800 nm. It may be not necessary to scan the full excitation range because the emission range eventually becomes very short; in practice, acquisition may be stopped at any chosen final excitation wavelength resulting in a “trapezoidal” rather than “triangular” array.

The FelixGX software controlling PTI fluorometers allows for programming the “action” (macro language script) to perform a series of acquisitions with simultaneously incrementing both the excitation wavelength and the “blue” end of the emission range. The *Fluorescence2D* software distribution includes an example action *Fluorescence2D.gxa*, which may be imported into FelixGX through the “*Action: (+): Automation Job: Import*” command. Because of relatively long acquisition times, it is important to ensure the Real-time Correction mode in FelixGX is enabled to compensate for a drift of the light source intensity (this is a default setting in *Fluorescence2D.gxa*).

Execution of this action results in a “triangular” dataset that may be exported from FelixGX via the *Export Group* command as a plain-text file that serves as an input for *Fluorescence2D*. The *Fluorescence2D* software package consists of two separate parts: (1) the data-conversion utility written in the Python language, and (2) the set of spectral-display programs operating in the MATLAB environment.

### Spectral data conversion

To make it possible to graph and analyze the triangular data, we need to bring the triangular matrix into a rectangular format by filling the “missing” shorter wavelength with a constant zero value. Setting intensity values to zero in the upper spectral triangle (Figure 1.B) does not create ambiguity because these values are only filled in the area above the diagonal where the data were *not* acquired. The Python program *pti2fluorescence2D.py* extracts spectral data from the FelixGX export file and performs the conversion. The *pti2fluorescence2D.py* saves the rectangular spectral data as three separate files: *spectral_intensity.txt* — contains a rectangular matrix of intensities; *emission_wavelengths_Xaxis.txt*, and *excitation_wavelengths_Yaxis.txt* — contain arrays of emission and excitation wavelengths corresponding to the columns and rows of the spectral intensity array. These data are used by the MATLAB part of the *Fluorescence2D* package to create spectral intensity plots. Currently, the data conversion is specific for the data exported from FelixGX (PTI). However, the plain text format of the converted data employed in *Fluorescence2D* enables easy development of additional converters for other fluorometers such as Horiba, Jasco, etc.

The FelixGX-exported data file contains the raw intensities followed by the corrected data either of which may be exported and analyzed. This choice is made using a command-line switch ‘*-raw*’ or ‘*-corr*’ in the *pti2fluorescence2D.py* call. Generally, it is the corrected data that one wants to use because all time- and instrument-dependent corrections have been automatically introduced by the FelixGX software. If the user wants to make customized corrections, the raw data may be utilized. Below is an example of the *pti2fluorescence2D.py* call on the command line of OS X and Linux computers to extract the corrected portion of the 2D data: *pti2fluorescence2D.py -corr FelixGX_exported_2D_data_in_triangle_form.txt*

### Display and analysis of the data

Baseline subtraction, representation of the 2D data and extraction of 1D slices in emission and excitation dimensions are implemented in the MATLAB environment (Mathworks). Capability of the MATLAB to perform vectorized computation as well as its efficient graphics tools allowed for creation of the most concise code easy for modification by the end user.

**Figure 3:**
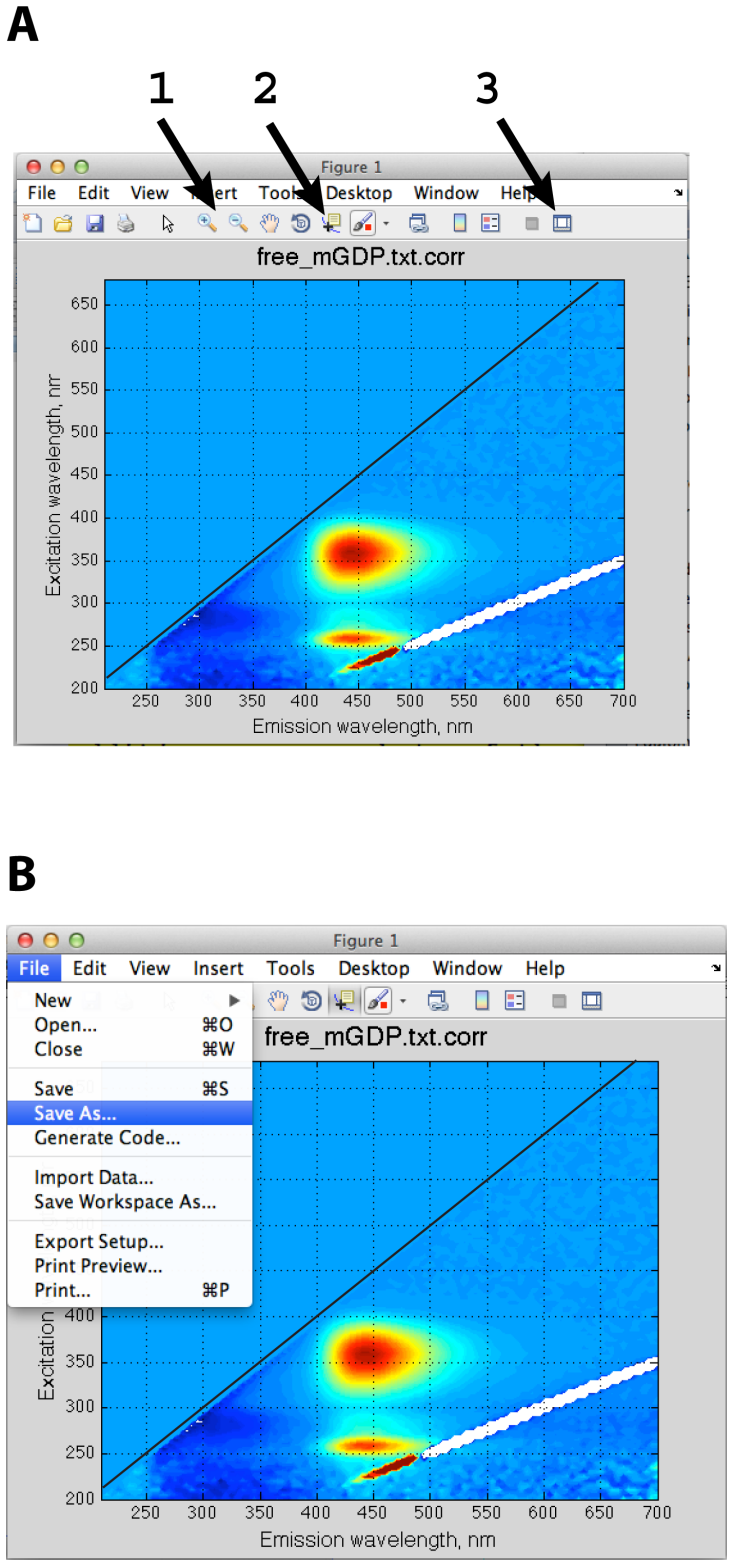
Manipulation of the graphic output of *Fluorescence2D*. The interactive tools of MATLAB allow for easy adjustment, inspection, and export of the graphics created by *Fluorescence2D*. In the panel **A**, arrows indicate locations of the zoom tool (**1**) used to enlarge the desired area, the data inspector tool (**2**) allowing to determine values of wavelength and intensity at any specific point, and the interactive graphics mode toggle (**3**) for adjustment of font sizes, labels, ticks, and colors. Export of the final image into a desired format is performed through *File:Save As* menu item as shown in Panel **B**.

The main control module of the *Fluorescence2D* MATLAB package is *fluorescence2Dmain.m* that the user would copy into the working folder with the data. The *fluorescence2Dmain.m* is a short script mainly composed of the user input section where all desired parameters are set. The *fluorescence2Dmain.m*, in turn, calls the library functions *fluorescence2Dplot.m* to create 2D graphs and *fluorescence2Dslice1D.m* to extract 1D slices at desired excitation or emission wavelengths. The typical workflow is to, first, adjust settings in *fluorescence2Dmain.m* and launch it from MATLAB command line. If the resulting graphs are not satisfactory, the settings in the *fluorescence2Dmain.m* are adjusted further and the script is re-launched. The choice between developing the graphical user interface versus the plain-text settings list was made in favor of the latter to make it simpler for the end user to modify and extend *Fluorescence2D* functionality. The interactive graphics tools of MATLAB allow easy modification of the obtained figures to obtain publication quality images (Figure 3). The detailed settings of *fluorescence2Dmain.m* are discussed below.

**Section “1. DATA SOURCE”** allows entering the name of the folder name created by *pti2fluorescence2D.py* as a value of *settings.dname* variable. It is recommended that a 2D spectrum of a buffer solution without addition of the fluorophores is also recorded and introduced in this section as *settings.baseline_name* variable to capture background fluorescence and instrumental artifacts. The baseline subtraction is turned on by *settings.baseline_correction = 'yes'*. This action may also be used for observation of a difference spectrum, for example, of two related samples or different time points in the reaction course. The baseline-corrected 2D matrix is also made accessible as a plain text format file *spectral_intensity_baseline_corrected.txt* in the folder *settings.dname*. The interval of intensities displayed as contours is defined by *settings.lowest_contour_percent* and *settings.highest_contour_percent* (full intensity scale is shown by 0% to 100%). These percentage values are specific for each 2D dataset; therefore, when working with multiple spectra, it is convenient to group these lines with the corresponding *settings.dname* entries and comment them out when a different dataset is displayed.

**Section “2. OPERATION MODE”** determines what type of a graph will be created: two-dimensional, one-dimensional excitation or emission spectra.

**Section “3. PLOTTING PARAMETERS”** contains *settings.graphics_export* variable controlling whether the graphs are only displayed on the screen or also exported in various graphics formats as well. Often, the 2D graphs contain signals of vastly different intensities and it may take several consecutive adjustments of the contour levels to emphasize the features of interest. Similarly, 1D slices may need adjustment of the wavelength range. If the user is not interested in saving all intermediate spectral views, the *settings.graphics_export* should be *'off'*. When the setting is '*on*', the four graphics files are created: (1) high-resolution image in the png format suitable for embedding in text documents and presentations; (2) the low-resolution png image for linking from the HTML documents; (3) the EPS format file and (4) the MATLAB figure (.fig) for interactive data inspection and adjustment of zoom level and other display parameters.

The next three sections describe parameters specific for the three operation modes:

**Section “4. 2D PARAMETERS”** controls appearance of the 2D graphs. The color map (scheme) is set via the parameter *settings.colormap_name*. The default colormap is blue to red; the user is referred to MATLAB Documentation for available colormap values. The contour plot is created with the number of contours set by *settings.N_colors*.

**Section “5. 1D EMISSION SLICE PARAMETERS”** sets the excitation wavelength for the desired emission spectrum (via *settings.emission_spectrum_excited_at*). The X-axis limits are set by the values of corresponding *settings.emission_spectrum_X_min* and *emission_spectrum_X_max*.

**Section “6. 1D EXCITATION SLICE PARAMETERS”** sets the emission wavelength for the desired excitation spectrum (*settings.excitation_spectrum_detected_at*) as well as X-axis limits (*settings.excitation_spectrum_X_min* and *settings.excitation_spectrum_X_max*).

In both one-dimensional display modes with the *settings.graphics_export* = *'on'*, the 1D data are also exported as a plain text array to enable further analysis by the user. If the requested emission or excitation wavelength does not exist in the spectral array, the *fluorescence2D* uses the nearest existing wavelength value.

Typically the user will start with *settings.mode = '2D'* and *settings.graphics_export* = *'off'* and initial contour levels set from 0% to 100%. Once the satisfactory values of the contour levels are found, the *settings.graphics_export* is set to *'on'* to direct *fluorescence2Dmain* to export graphic files. With this setting, the MATLAB figure is saved; in addition, the user has an option to export any of the three graphic files (or all of them). The values of the current contour levels are included in the file names. Therefore, subsequent re-runs of the script with different contour levels will generate a family of files corresponding to different contour settings to allow for easy comparison.

In the next step, the 1D slices at desired wavelengths may be created as well in a separate runs of *fluorescence2Dmain* with *settings.mode = '1D emission slice'* or *settings.mode = '1D excitation slice'* (again, use *settings.graphics_export* = *'off'* for trial runs to prevent exporting the graphics). When the graphics are exported, the emission and excitation wavelengths are included in the file name. Sometimes, the default axis labels and ticks are unsatisfactory and custom adjustments are needed. In this case, the user would edit the corresponding MATLAB figure using the MATLAB interactive graphics toolbar (Figure 3). The result may be saved as another MATLAB figure (extension “fig”) for future adjustments and also into other graphics formats via the *File:Save As* menu.

### Installation of the software

The steps outlined below describe the installation procedure on OS X and Linux systems:

**Step 1.** Unpack the *Fluorescence2D.zip* in any convenient location *location/*. Add the following line to your.cshrc file: *set path = ($path. location/Fluorescence2D)*. Type *pti2fluorescence2D.py* on the command line to see a standard printout with usage notes indicating that the converter works.

**Step 2.** The MATLAB portion of Fluorescence2D consists of fluorescence2Dslice1D.m, fluorescence2Dplot.m, and fluorescence2Dmain.m. To make them accessible for MATLAB, go to Environment tab in MATLAB toolbar, Set Path: Add folder and point at location/Fluorescence2D.

**Step 3.** Copy *fluorescence2Dmain.m* into the folder with your data and edit it to insert desired settings.

## Materials and Methods

Mant-GDP ((2'-(or-3')-O-(N-Methylanthraniloyl) Guanosine 5'-Diphosphate, Disodium Salt) was purchased from Life Technologies (Cat # M-12414). The human H-Ras protein, residues 1–166, was prepared as described previously [11] and loaded with mant-GDP according to [12]. The sample buffer contained 20 mM Hepes pH 7.2, 150 mM NaCl, 1 mM MgCl_2_, 1 mM DTT, and 0.1% NaN_3_. The fluorescence spectra were obtained using PTI QM40 instrument with 5 nm excitation and emission slit widths, in the wavelength ranges from 200 to 700 nm with the 5 nm step and 0.05 second integration time.

## Results and Discussion

To illustrate an application of the *Fluorescence2D* software for analysis of a sample containing multiple fluorophores, I obtained a 2D fluorescence spectrum of H-Ras protein bound to mant-GDP. The triangular datasets were recorded, first, for the buffer solution alone followed by the 1 µM protein solution. The time to collect one triangular 2D dataset was 23 minutes (compared to 50 minutes per a corresponding rectangular array).

**Figure 4:**
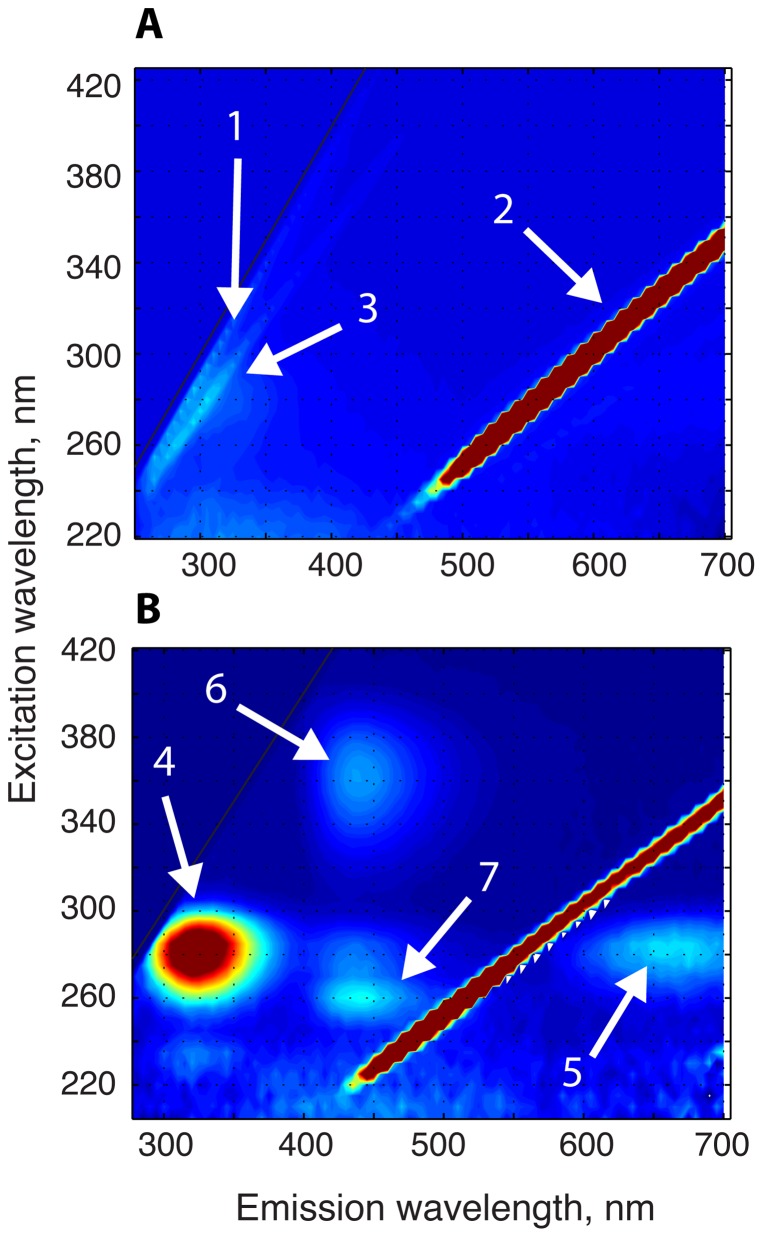
Two-dimensional spectra of H-Ras in the complex with mant-GDP. (Panel **A**) The 2D spectrum of the buffer solution (without baseline subtraction) is shown contoured between 0 and 10% of total intensity (blue to red). The first order reflection, **1**; the second order reflection, **2**; Raman scattering, **3**. The black solid line separates the lower area with experimental data from the upper area added to make the dataset rectangular for visualization. (Panel **B**) The 2D spectrum of H-Ras in the complex with mant-GDP. The spectrum of the buffer solution (from Panel **A**) was subtracted as a baseline; the resulting graph was contoured from 3 to 13% of total intensity scale. Tyrosine fluorescence, **4**; its second order reflection, **5**; mant-GDP emission, **6** and **7**. Spectra in both panels were “zoomed in” using interactive MATLAB graphics tools. The original and processed experimental data along with the corresponding *fluorescence2Dmain.m* script are included with the *Fluorescence2D* package in the *sample_data/*folder.

The Figure 4.A shows a corrected 2D spectrum of the buffer solution. It is instructive to view the baseline spectra prior to the sample measurement to appreciate existing spectral artifacts. This spectrum contains strong artifacts originating from the grating of the emission monochromator around λ_em_ = λ_ex_ (the first-order reflection, **1**) and λ_em_ =  2λ_ex_ (the second-order reflection, **2**). Both of these reflections arise due to small amount of scattered excitation light entering emission channel because protein solutions often have small particles due to spontaneous protein aggregation [1]. The contribution from the first-order reflection is reduced by ensuring that the emission range always begins at a few nanometers longer wavelength than the current excitation wavelength. However, the intensity of the second-order reflection remains sufficiently large to render automatic scaling of the 2D spectra impractical; therefore, the *Fluorescence2D* relies on the manual adjustment of contour levels by the user until the desired spectral appearance of the spectrum is achieved. Another typical spectral artifact is Raman scattering from water observed as a ridge running at a small angle to the first order reflection (labeled as **3** in the panel **A**).

The Figure 4.B demonstrates the 2D spectrum of Ras complex with mant-GDP obtained with the baseline subtraction. The second-order reflection remains large due to its very high total intensity (leading to saturation of the detector); the contributions from the first order reflection and Raman scatter were successfully removed. The strongest fluorescence in this sample originates from tyrosines (**4**) of Ras observed as a peak at 280 nm excitation and 330 nm emission wavelengths. Another peak (**5**) at 280 nm excitation wavelength and doubled emission wavelength (360 nm) is an artifact of the monochromator grating: the second-order reflection of tyrosine fluorescence. The mant group of GDP fluoresces at 440 nm excited at 360 nm and at 260 nm, which are likely due to S_0_ to S_1_ and S_0_ to S_2_ transitions, respectively. This sample displays intensity of the tyrosine peak much greater than that of mant-GDP indicating incomplete exchange of the endogenous GDP for mant-GDP in Ras.

**Figure 5:**
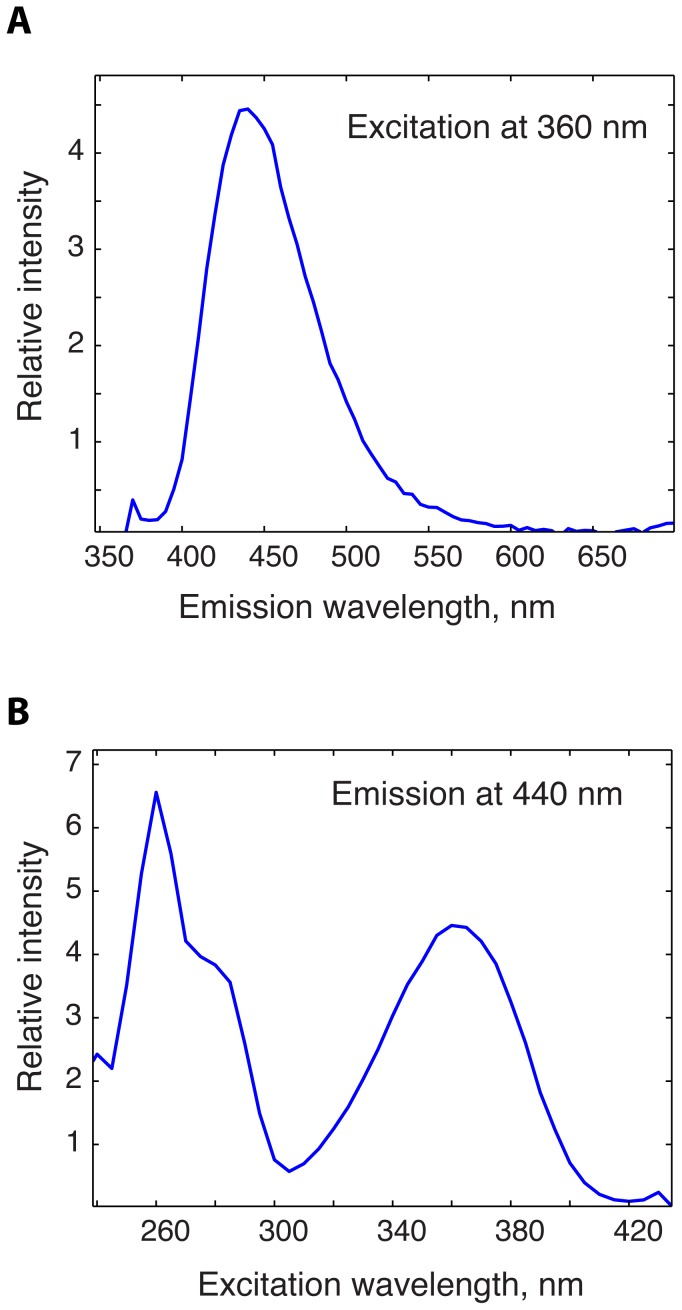
One-dimensional slices of the 2D spectrum extracted at specified emission and excitation wavelengths. The 1D spectra were extracted from the full 2D spectrum at the excitation wavelength of 360(**A**), and at the emission wavelength of 440 nm (**B**), respectively.

The primary use of the two-dimensional fluorescence spectra is to enable making an informed selection of the wavelengths for the one-dimensional emission or excitation scans such that interference between fluorophores is reduced. The *Fluorescence2D* allows for extraction of 1D slices out of the 2D spectral matrix at desired wavelengths as if the one-dimensional experiments were carried out (Figure 5). Once the optimal wavelengths are selected, additional one-dimensional experiments may be performed with increased sensitivity and resolution.

In summary, acquisition of the two-dimensional fluorescence spectra is accelerated by using the “triangular” acquisition protocol. The *Fluorescence2D* software enables display and analysis of such triangular two-dimensional fluorescence spectra, and extraction of one-dimensional slices simulating corresponding excitation and emission scans. The *Fluorescence2D* package and sample data are freely available from http://lineshapekin.net/#Fluorescence2D.
